# Clinical Significance of Exposure to Microplastics and the Risk of Diabetes Mellitus in Humans: A Systematic Review

**DOI:** 10.7759/cureus.110956

**Published:** 2026-06-16

**Authors:** Abhishek Vadher, Swati Baraiya, Bobbadi Gajendra Siva Krishna Pavan Kumar, Sajith Silva, Mehwish Zeb, Utsav R Thakkar, Kesava Manikanta Achuta, Apoorv Tiwari, Pranay Gupta, Andrew Zazaian, Sujata Kambhatla

**Affiliations:** 1 Internal Medicine, Garden City Hospital, Garden City, USA; 2 Family Medicine, Bombay Hospital and Medical Research Centre, Mumbai, IND; 3 Internal Medicine, Vedant Multispeciality Hospital, Ahmedabad, IND; 4 Endocrinology, University of Minnesota, Minneapolis, USA

**Keywords:** bottled water, diabetes mellitus, microplastics, nanoplastics, obesity

## Abstract

Microplastics and nanoplastics are pervasive environmental contaminants present in food, water, and air. Many studies have shown bioaccumulation of microplastics in human organs such as the lungs, colon, blood, and even placental tissue. Microplastics are potentially linked to many diseases such as colorectal cancer, lung cancer, obesity, metabolic syndrome, coronary artery disease, and dementia. There is also a potential link between microplastic exposure and diabetes. The incidence of diabetes is rapidly increasing across all countries globally. We conducted this systematic review to study the potential link between microplastic exposure and diabetes or adverse glycemic control in diabetics. A systematic search strategy was used, as per the Preferred Reporting Items for Systematic Reviews and Meta-Analyses 2020 guidelines. We searched PubMed, Embase, Scopus, Google Scholar, and Cochrane. A total of 1,239 records were identified, of which 141 duplicates were removed, and 1,098 articles were screened. A total of six studies were included in the results. A meta-analysis could not be conducted as all articles were heterogeneous in exposure to microplastics, methods, etc. Two studies directly measured microplastic levels, one in urine samples of pregnant patients and another in blood serum. Both studies found a statistical significance of higher microplastic levels and worsening of diabetes control. Two studies assessed exposure by proxy-based methods and questionnaires. Participants consisted of people who consumed food in heated plastics and plastic bottled water. These studies found a correlation of higher HbA1c and increased risk of diabetes mellitus, with one study explicitly mentioning increased risk of type 2 diabetes mellitus. Two ecological studies where microplastic exposure was obtained from environmental data showed a higher prevalence of diabetes in populations where exposure was higher. While our data indicate an association between microplastic exposure and diabetes, the absence of large, prospective cohort studies currently precludes the establishment of a causal relationship. As plastic pollution accelerates globally, addressing this critical knowledge gap through rigorous longitudinal research is essential.

## Introduction and background

Over the past several decades, the prevalence of diabetes and obesity in the United States has increased dramatically, reflecting a complex interplay of genetic, lifestyle, and environmental factors. According to data from the Centers for Disease Control and Prevention (CDC), approximately 9% of U.S. adults had diabetes in the early 2000s, rising to nearly 14% between 2017 and 2020, a 55% increase over two decades, with the current burden of close to 38 million among the U.S. population [1]. During a similar timeframe, obesity rates have shown an even more pronounced rise, from roughly 13% in the 1960s to nearly 40% in recent years, with a notable plateau observed in the last decade [2,3]. While excessive caloric intake, sedentary behavior, and genetic predisposition are well-established contributors to these metabolic disorders, emerging evidence suggests that environmental factors, particularly microplastics and nanoplastics, may potentially play an increasingly significant role in metabolic dysregulation. Microplastics, defined as particles smaller than 5 mm, and nanoplastics, smaller than 1 µm, are generated through the breakdown of larger plastic materials from packaging, containers, personal care products, and various synthetic items. Their omnipresence in food, water, air, and consumer products has raised concerns regarding their potential contribution to chronic metabolic disorders, including type 2 diabetes and obesity.

Human exposure to microplastics occurs through multiple everyday routes, making it nearly unavoidable. Americans are estimated to ingest and inhale between 74,000 and 121,000 microplastic particles annually, with the highest exposures arising from bottled water, heated food in plastic containers, takeout packaging, processed foods, and indoor dust contaminated with synthetic fibers [4]. For instance, 1 L of bottled water may contain up to 240,000 plastic particles, demonstrating the magnitude of exposure through routine consumption [5]. Inhalation of microplastics from household dust is another significant pathway, particularly in indoor environments containing synthetic textiles, carpets, and upholstered furniture. Clothing made from polyester, nylon, and acrylic releases fibers during wear and laundering, contributing further to airborne microplastic exposure. Additional exposure sources include plastic kitchenware, such as cutting boards, utensils, and non-stick cookware, which release particles when exposed to heat, friction, or repeated use. Environmental sources, including tire wear, road dust, and urban pollution, further exacerbate exposure, allowing microplastics and nanoplastics to enter the bloodstream and potentially accumulate in various organs [6,7]. The extremely small size of these particles enables their infiltration into cells and tissues, raising concerns about both immediate and long-term health effects, particularly metabolic disturbances.

Accumulating evidence indicates that microplastics exert their harmful effects through multiple biological mechanisms, many of which are directly linked to metabolic dysfunction. These particles and their associated chemical additives, including bisphenol A (BPA) and phthalates, can potentially disrupt endocrine function and interfere with insulin signaling, thereby reducing insulin sensitivity and contributing to hyperglycemia and type 2 diabetes. Additionally, certain plastic-associated compounds may act as obesogens, promoting adipogenesis, altering energy metabolism, and favoring fat accumulation over energy expenditure. Microplastics may induce chronic low-grade inflammation and oxidative stress, both of which are central to the development of insulin resistance, metabolic syndrome, and weight gain. Disruption of the gut microbiome is another critical mechanism; exposure to microplastics has been potentially linked to reduced populations of beneficial gut bacteria, increased intestinal permeability, and the onset of “leaky gut,” all of which are strongly associated with obesity and impaired glucose regulation. At the cellular level, microplastics and nanoplastics may compromise mitochondrial function, reduce cellular energy efficiency, trigger oxidative damage, and impair cell proliferation. These multifaceted impacts highlight the potential for microplastics to act as environmental contributors to the rising prevalence of metabolic disorders.

Given the extensive and ubiquitous exposure to microplastics and their demonstrated biological effects, there is an urgent need for comprehensive research to further elucidate their role in the development of obesity, diabetes, and related metabolic disorders. The potential mechanisms, ranging from endocrine disruption and oxidative stress to mitochondrial dysfunction and gut microbiome alterations, highlight the complexity of interactions between environmental pollutants and human health. Public awareness of microplastic exposure remains limited, despite the substantial evidence of their contribution to chronic disease. Understanding these connections is critical not only for advancing scientific knowledge but also for informing public health interventions aimed at reducing exposure, mitigating risk, and addressing the ongoing rise in metabolic disorders. As society continues to rely heavily on plastic products, the identification and management of microplastic exposure emerge as a vital component of preventative health strategies in the 21st century.

This systematic review aimed to evaluate human studies assessing measured or proxy microplastic exposure in relation to diabetes, gestational diabetes, insulin resistance, HbA1c, fasting glucose, or impaired glucose tolerance.

## Review

Methodology

The current study was conducted as per the Preferred Reporting Items for Systematic Reviews and Meta-Analyses (PRISMA) 2020 guidelines. A literature search was performed in PubMed, Embase, Cochrane, Scopus, and Google Scholar. The detailed search strategy is provided in the Appendices.

A total of 1,239 articles were obtained, of which 141 duplicate articles were removed, and 1,098 articles were screened. Two independent authors (AV and SB) performed the screening. Disagreements were resolved by a third author (AT). A total of 18 articles were selected, and full manuscripts were obtained. Three authors (AV, AT, and SB) conducted the full-text screening. Of the 18 full-text articles assessed for eligibility, 12 were excluded. Five articles were excluded because they did not evaluate documented human microplastic exposure and instead focused on plastic additives, endocrine-disrupting chemicals, or mechanistic toxicology pathways. Three articles were excluded because they were reviews, commentaries, or policy papers without original human data. Two articles were excluded because they involved animal, experimental, or laboratory models rather than human participants. One article was excluded because it was a study protocol without reported outcomes. One article was excluded because it did not provide extractable quantitative data regarding the association between microplastic exposure and diabetes-related outcomes. A total of six articles were included for the final review. Data extraction was done by two authors (AV and AT), and any discrepancy was resolved by a third author (SB). Study design, population characteristics, sample types, microplastic exposure assessment, primary and secondary outcomes, and biological mechanisms were extracted from the articles. Due to the heterogeneity of study design, exposure measurement, outcomes definition, and effect estimates, quantitative pooling was not possible, and a meta-analysis was not performed.

Study Selection Criteria

Inclusion criteria: Studies exclusively on humans with documented microplastic exposure. Exposure was evaluated by serum or urinary levels of microplastics, indirect exposure through questionnaires, or environmental exposure. Studies reporting on diabetes mellitus incidence and prevalence with quantitative data. Case-control, cohort, and observational studies were considered for inclusion.

Exclusion criteria: Studies on animals, in vitro studies, reviews, case reports, and editorials were excluded. Disagreements were resolved by consensus. The exact search strategy is mentioned in the Appendices. PICO components are presented in Table 1.

**Table 1:** PICO components.

P (Population)	I (Intervention or Exposure)	C (Comparator)	O (Outcome)
Adults and/or children from the general population or occupationally exposed populations. No restrictions on sex, ethnicity, or geographic location	Exposure assessed through (1) direct biomarker-based assessment (microplastics measured in blood, urine, serum, or other biological samples); (2) individual-level proxy exposure indicators (e.g., bottled water consumption, plastic container use, hot food in plastic packaging); and (3) ecological/environmental exposure estimates (e.g., regional marine or environmental microplastic burden)	Individuals with low microplastic exposure, no detectable exposure, or those in the lowest exposure category/quantile in dose-response studies	Primary outcomes included diabetes mellitus (type 1, type 2, and gestational diabetes, where applicable), diabetes incidence/prevalence, and new-onset diabetes. Secondary outcomes included impaired glucose tolerance, insulin resistance, fasting plasma glucose abnormalities, and elevated HbA1c levels

The methodological quality and risk of bias of the six included studies were evaluated independently by two reviewers (AV, SB), with disagreements resolved through discussion with a third reviewer (AT). Given the heterogeneity of study designs, i.e., cross-sectional, case-control, and ecological, no single validated tool was applicable to all studies. Accordingly, the Joanna Briggs Institute Critical Appraisal Checklist was used for case-control and cross-sectional studies. For ecological studies, the Newcastle-Ottawa Scale was employed. The adapted tools retained the core domains of selection, comparability, and outcome/exposure assessment, with modifications to accommodate the specific design features of each study type. Each study was rated as having low, moderate, or high risk of bias across the assessed domains, and an overall risk-of-bias judgment was assigned. The risk of bias was assessed by two independent authors (MZ and URT). Risk assessment of each study is documented in Table 2.

**Table 2:** Risk of bias assessment. JBI = Joanna Briggs Institute; OGTT = oral glucose tolerance test; GIS = geographic information system

Study	Design	Tool used	Main strengths	Main bias concerns	Overall risk
Alharbi et al. [8]	Cross-sectional	JBI cross-sectional	Large sample; biochemical outcomes measured objectively; clear population	Exposure was proxy/self-reported plastic use with hot food; no direct microplastic measurement; no classification of plastic type or emitted chemicals	Moderate
Ma et al. [9]	Cross-sectional	JBI cross-sectional	Large sample; urinary microplastics measured directly; standardized OGTT; robust adjusted models	Cross-sectional design; urine reflects recent exposure, not long-term exposure; measurement protocols for urinary microplastics still evolving	Low to moderate
Felek et al. [10]	Case-control	JBI case-control	Direct serum microplastic measurement; contamination-control procedures; regression adjustment	Major age imbalance between diabetic and control groups; small sample; reverse causality possible because diabetes may influence microplastic accumulation	Moderate
Ponnana et al. [11]	Ecological abstract	Newcastle-Ottawa Scale	Objective public datasets; census-tract-level exposure/outcome linkage; adjusted modeling with multiple socioeconomic/environmental features	Ecological fallacy; no individual-level microplastic exposure; abstract-only data; temporality unclear	High
Makwana et al. [12]	Ecological abstract	Newcastle-Ottawa Scale	GIS-based marine microplastic exposure; adjusted quasi-Poisson regression; population-weighted analysis	Ecological fallacy; county-level exposure assignment; abstract-only data; limited covariate detail; no individual exposure measurement	High
Dolcini et al. [13]	Cross-sectional	JBI cross-sectional	Very large nationally representative sample; logistic regression; adjusted for several covariates	Bottled-water use was only a proxy exposure; self-reported disease data; no exact bottled water quantity; dietary confounding not directly measured	Moderate to high

**Figure 1:**
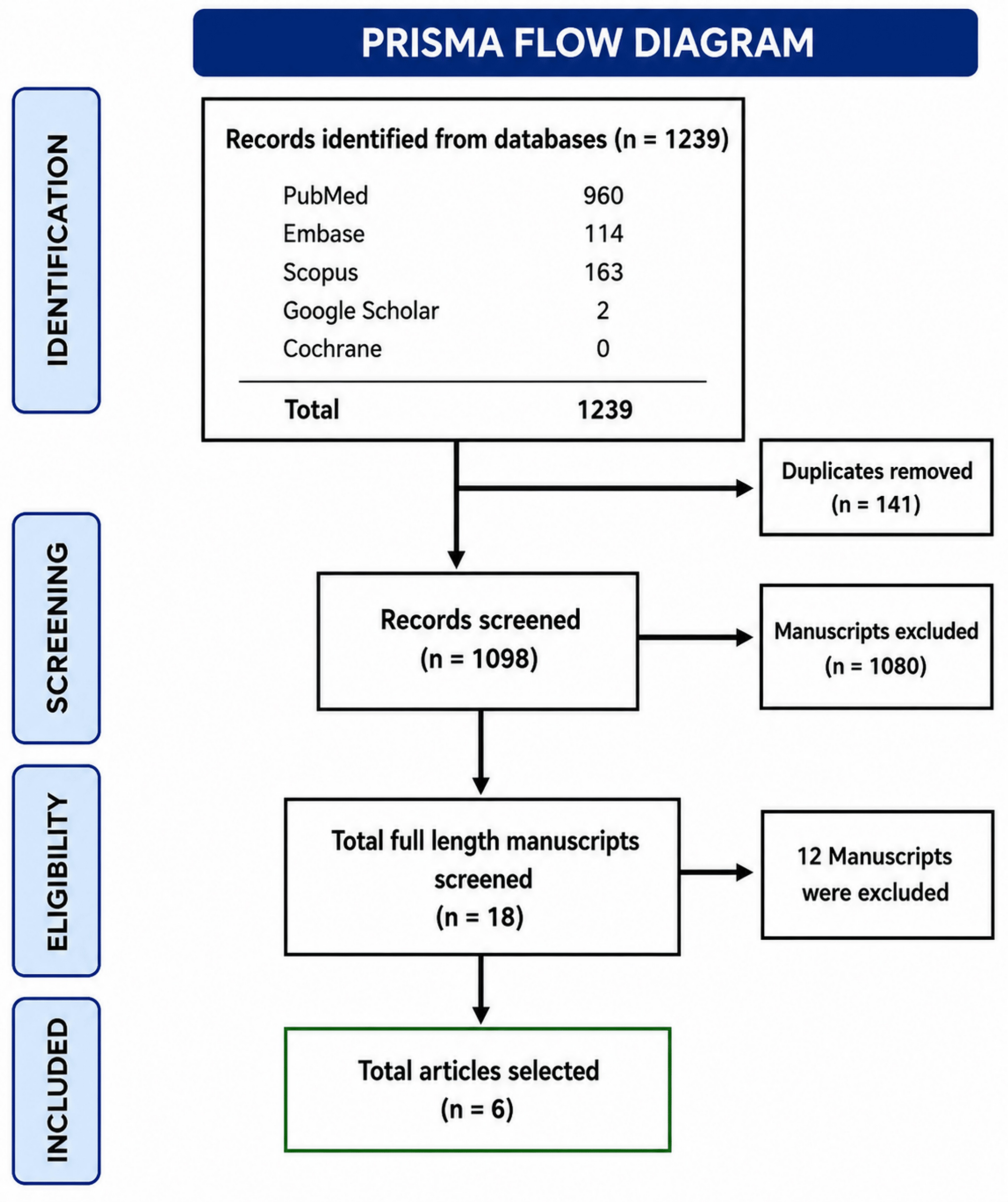
Preferred Reporting Items for Systematic Reviews and Meta-Analyses (PRISMA) flow diagram.

PRISMA flowsheet is shown in Figure 1.

Results

A total of six studies met the inclusion criteria. Studies consisted of cross-sectional, case-control, and ecological study designs conducted across different populations.

Direct Biomarker Studies

Two studies utilized direct biomarker-based assessment of microplastic exposure. In a cross-sectional study of 3,775 pregnant women, higher urinary microplastic concentrations were significantly associated with increased fasting plasma glucose (increase of 1.53-2.47 mg/dL per interquartile range, p < 0.01) and elevated oral glucose tolerance test values [9]. In a case-control study, diabetic patients demonstrated significantly higher serum microplastic levels compared to healthy controls (3.14 ± 1.30 vs. 1.50 ± 0.89 µg/mL, p < 0.001), with positive correlations observed between microplastic levels and both HbA1c and fasting glucose (p = 0.001) [10].

Behavioral/Proxy Exposure Studies

Two studies assessed proxy-based exposure. Frequent use of plastics with hot food was associated with significantly higher HbA1c levels among pregnant women (p < 0.05) [8]. Higher bottled water consumption was associated with an increased risk of diabetes (odds ratio (OR) = 1.09, 95% confidence interval (CI) = 1.01-1.18) [13].

Ecological/Environmental Studies

Two ecological studies evaluated environmental exposure at the population level. A positive correlation was observed between environmental microplastic concentration and diabetes prevalence (r = 0.30, p < 0.001) [11]. Similarly, regions with higher marine microplastic levels demonstrated an 18% higher prevalence of diabetes (prevalence ratio (PR) = 1.18, 95% CI = 1.13-1.23, p < 0.001) [12]. The results of the studies are mentioned in Table 3.

**Table 3:** Summary of study designs, exposure assessments, and clinical outcomes regarding microplastics and diabetes and secondary outcomes. LC/GC = liquid chromatography/gas chromatography; MS = mass spectrometry; HPLC = high-performance liquid chromatography; SEM-EDS = scanning electron microscopy-energy dispersive spectroscopy; FPG = fasting plasma glucose; OGTT = oral glucose tolerance test; TSH = thyroid-stimulating hormone; NCEI = National Centers for Environmental Information; g-comp = g-computation; qWQS = quantile-based weighted quantile sum; ANOVA = analysis of variance; CAD = coronary artery disease; MML = marine microplastic level; GIS = geographic information system; PR = prevalence ratio; OR = odds ratio; CI = confidence interval

Study	Study design, country, year	Population characteristics	Sample type, microplastic exposure assessment	Types of microplastics and detection methods	Primary outcomes	Secondary outcomes	Statistical model
Felek et al. [10]	Observational case-control study, Turkey, 2025	50 diabetic patients and 50 healthy controls	Serum microplastic measurement	Detected using LC/GC-MS/MS and HPLC techniques	Higher microplastic levels in diabetics (3.14 vs. 1.50 µg/mL, p < 0.001). Positive correlation with HbA1c and glucose (p = 0.001)	-	Multivariate linear regression
Ma et al. [9]	Cross-sectional study, China, 2022–2023	3,775 pregnant women (24–28 weeks’ gestation)	Urinary microplastic measurement (biomarker-based )	Polyamide, polypropylene, polyvinyl chloride, and polyethylene detected via microscopy, SEM-EDS, and staining methods	FPG: A one-quartile increase in microplastic mixture exposure was associated with a 2.95 mg/dL increase in FPG (95% CI = 1.89, 4.02; p < 0.01) in the g-comp model and a 3.85 mg/dL increase (95% CI = 2.55, 5.15; p < 0.01) in the gWQS model. 1-hour OGTT: A one-quartile increase in microplastic mixture exposure was associated with a 4.22 mg/dL increase (95% CI = 2.47, 5.96; p < 0.01) in the g-comp model and a 3.79 mg/dL increase (95% CI = 1.88, 5.70; p < 0.01) in the gWQS model. 2-hour OGTT: A one-quartile increase in microplastic mixture exposure was associated with a 2.60 mg/dL increase (95% CI = 1.30, 3.91; p < 0.01) in the g-comp model and a 2.41 mg/dL increase (95% CI = 0.86, 3.45; p < 0.01) in the gWQS model	-	Multivariable linear regression, Bayesian kernel machine regression, g-computation (g-comp), and qWQS regression
Alharbi et al. [8]	Cross-sectional study, Saudi Arabia, 2009–2011	740 healthy pregnant women (first trimester)	Behavioral/Proxy exposure (use of plastics with hot food)	Not directly measured; exposure inferred from plastic use frequency	↑ HbA1c (higher in daily users, p < 0.05)	↑ TSH, ↑ homocysteine; ↓ vitamin E, Zn, and Se	Simple linear regression for each biochemical outcome, with frequency of plastic use with hot food as the independent variable, adjusted only for age
Dolcini et al. [13]	Cross-sectional study, Italy; 2021	45,597 adults (national survey)	Bottled water consumption (proxy exposure)	No direct detection; microplastic exposure inferred from bottled water (~240,000 particles/L)	↑ Diabetes risk (OR = 1.09, 95% CI = 1.01–1.18). Diabetes: OR = 1.09, CI = 1.01-1.18, p-value = 0.005		Multivariable logistic regression (binary outcome)
Ponnana et al. [11]	Ecological study, USA, 2025	555 coastal census tracts (population level data )	Environmental microplastic exposure (linked datasets)	Microplastic concentration from NCEI datasets; modeled exposure	↑ Diabetes prevalence (r = 0.30; p < 0.001)	↑ Hypertension, ↑ stroke (significant ANOVA results). High BP: r = 0.24, F = 7.351, p < 0.001. Stroke: r = 0.26, F = 8.798, p < 0.001	Multivariable Poisson regression
Makwana et al. [12]	Ecological study, USA, 2015–2020	Coastal county populations	Marine microplastic concentration (ocean data)	Environmental levels categorized (low → very high) via GIS analysis	↑ Diabetes prevalence (PR = 1.18, p < 0.001). Type 2 diabetes: 13.03% in very high MML counties, 11.23% in low MML counties; adjusted PR = 1.18 (1.13-1.23)	↑ CAD, ↑ stroke prevalence. CAD: 8.94% in very high MML counties, 7.43% in low MML counties; adjusted PR = 1.07 (1.03-1.11). Stroke: 4.17% in very high MML counties, 3.51% in low MML counties; adjusted PR = 1.09 (1.05-1.13)	Population‑weighted quasi‑Poisson regression

Discussion

To our knowledge, this is the first systematic review to evaluate the clinical significance of microplastic exposure and its association with the risk of diabetes mellitus in humans. Across the six included studies, the available evidence suggests a positive association between higher microplastic exposure and diabetes-related outcomes. Regardless of the type of exposure, quantified by serum and urine levels, or estimated through proxy behaviors and environmental datasets, high levels of microplastic exposure were associated with increased fasting blood glucose levels, impaired oral glucose tolerance tests, higher HbA1c levels, and increased prevalence and risk of diabetes mellitus. However, the evidence is small, heterogeneous, and at high risk of bias.

Biological Mechanisms and Plausibility

The mechanism of microplastics and nanoplastics potentially causing diabetes is multifactorial, and many different mechanisms of action have been proposed.

Endocrine disruption: Microplastics may absorb or contain chemical additives, such as BPA and phthalates, which are known to cause endocrine disruption, also referred to as endocrine-disrupting chemicals. These chemicals can interfere with insulin signaling pathways, reduce peripheral insulin sensitivity, and promote adipogenesis, eventually creating a foundation for Insulin resistance [14-16].

Oxidative stress and systemic inflammation: The physical presence of microplastics in tissues is known to trigger an immune response, which leads to chronic, low-grade systemic inflammation and oxidative stress [17]. The inflammatory state leads to cellular toxicity, particularly in pancreatic beta cells, which are highly susceptible to oxidative stress, and this mechanism is a core pathophysiological driver of diabetes [18].

Gut microbiota dysbiosis: Ingested microplastics directly interact with the gastrointestinal tract. Current evidence suggests that microplastics can alter the composition of the gut microbiome and reduce beneficial taxa. Microplastics also increase intestinal permeability (“leaky gut”) [19,20]. This endotoxemia further exacerbates systemic inflammation and impairs glucose homeostasis [21].

Cellular toxicity and epigenetic modifications: The incredibly small size of these particles allows them to cross cellular membranes. This can potentially cause direct mitochondrial dysfunction, reducing cellular energy efficiency, and inducing epigenetic modifications that can predispose individuals to metabolic dysregulation [22].

Microplastics are associated with increased risk of obesity and metabolic disease by inhibition of lipolysis and promotion of lipid accumulation [23-25]. As obesity is strongly associated with type 2 diabetes mellitus, microplastic exposure is associated with an increased risk of type 2 diabetes mellitus through metabolic syndrome and insulin resistance as plausible mediators.

The studies utilizing direct biomarker assessments provide the most compelling evidence for a direct physiological impact. Felek et al. [10] demonstrated that serum microplastic levels were more than twice as high in diabetic patients compared to healthy controls, with a strong positive correlation to HbA1c. Similarly, Ma et al. [9] found that even small incremental increases (one-quartile) in urinary microplastic mixtures were associated with significant elevations in fasting plasma glucose and oral glucose tolerance test values among pregnant women. This raises critical concerns about the role of microplastics in the development of gestational diabetes.

Proxy and ecological studies further corroborate these systemic findings on a population level. Dolcini et al. [13] established a 9% increased risk of diabetes (OR = 1.09) linked to high bottled water consumption, a known major source of ingested microplastics. Furthermore, the study by Ponnana et al. [11] and Makwana et al. [12] revealed that populations residing in areas with high environmental and marine microplastic concentrations exhibited a significantly higher prevalence of diabetes (PR = 1.18). Further, there are increased rates of hypertension, stroke, and coronary artery disease in these populations. This suggests that environmental microplastic pollution is not just an ecological crisis but a pressing determinant of cardiometabolic public health. Proxy-based studies are prone to recall bias, and ecological studies provide evidence at the population level and not the individual level. These study designs can potentially create a bias.

A few studies on mice have also found a strong correlation between exposure to microplastics and nanoplastics and increased risk of diabetes mellitus [26,27]. Potential mechanisms include dysregulated insulin secretion and insulin resistance, oxidative stress, and glucose intolerance [26].

Limitations

Although this systematic review is strong due to its comprehensive approach, there are many limitations which should be acknowledged. First, all the studies were cross-sectional or ecological in nature, making it difficult to establish causality. Second, the measurement of microplastics was heterogeneous across the studies. While studies utilizing liquid chromatography/gas chromatography-mass spectrometry (MS)/MS or scanning electron microscopy-energy dispersive X-ray spectroscopy provide precise quantification, proxy studies relying on questionnaires (e.g., frequency of plastic use with hot food or bottled water consumption) are inherently subject to recall and confounding biases. Lastly, the total number of studies in our review was fairly low for a systematic review. There are many confounding risk factors for diabetes mellitus such as a positive family history, diet, obesity, body mass index, socioeconomic status, physical activity, medication use, other medical conditions, and lifestyle. These variables were not thoroughly described in the included studies. The lack of these details adds a significant limitation to our systematic review.

Future directions

Future research must be conducted with a large patient population with a prospective cohort design to establish temporality and a more substantial link between microplastic exposure and diabetes incidence and glucose management. There is also a critical need to standardize the methods used for microplastic and nanoplastic detection from blood serum and urine samples.

Until substantial and more concrete evidence is established, in favor of public health, we advise people to avoid heating food in plastic containers and reduce the use of single-use bottled water.

## Conclusions

Current evidence shows a potential link between exposure to microplastics and the rising incidence of diabetes mellitus. The probable mechanisms of action include chronic inflammation, endocrine disruption, and cellular toxicity. Bioaccumulation of microplastics in human bodies may carry a silent threat to human health. The link between microplastics and diabetes needs to be concretely established by future prospective cohort research in humans.

## Appendices

Search strategy

Date of search: 3/3/26

1. PubMed: 960 hits

("Microplastics"[Mesh] OR "Plastics"[Mesh] OR microplastic* OR nanoplastic* OR "plastic particles" OR "plastic debris") AND ("Diabetes Mellitus"[Mesh] OR "Diabetes Mellitus, Type 2"[Mesh] OR "Diabetes Mellitus, Type 1"[Mesh] OR diabetes* OR "insulin resistance" OR hyperglycemia OR HbA1c) AND (humans[MeSH Terms])

2. Embase: 114 hits

('microplastic'/exp OR microplastic* OR nanoplastic* OR 'plastic particle' OR 'plastic debris') AND ('diabetes mellitus'/exp OR 'type 2 diabetes mellitus'/exp OR 'type 1 diabetes mellitus'/exp OR diabet* OR 'insulin resistance'/exp OR hyperglycemia) AND [humans]/lim

3. SCOPUS: 163 hits

TITLE-ABS-KEY (microplastic* OR nanoplastic* OR "plastic particles" OR "plastic debris") AND TITLE-ABS-KEY (diabetes OR "insulin resistance" OR hyperglycemia OR HbA1c)

4.     Google Scholar: 2

"microplastics" AND "diabetes mellitus" (Filter: in the title of the article)

5. Cochrane: 0

(microplastic* OR micro-plastic* OR nanoplastic* OR nano-plastic* OR "plastic particle*" OR "plastic debris" OR "plastic pollution" ) AND ( "diabetes mellitus" OR diabetes OR "type 1 diabetes" OR "type 2 diabetes" OR T1D OR T2D OR hyperglycemia OR "fasting plasma glucose" OR FPG OR HbA1c OR "glycated hemoglobin" OR "insulin resistance" OR HOMA-IR OR "glucose metabolism" OR "glucose intolerance" OR "gestational diabetes" OR GDM)
